# Prediction of Pathological Subthalamic Nucleus Beta Burst Occurrence in Parkinson's Disease

**DOI:** 10.1002/mds.70076

**Published:** 2025-10-03

**Authors:** Bahman Abdi‐Sargezeh, Sepehr Shirani, Abhinav Sharma, Tao Liu, Alexander Green, Harith Akram, Ludvic Zrinzo, Patricia Limousin, Tom Foltynie, Timothy Denison, Huiling Tan, Vladimir Litvak, Simon Little, Philip A. Starr, Ashwini Oswal

**Affiliations:** ^1^ MRC Brain Network Dynamics Unit University of Oxford Oxford UK; ^2^ Nuffield Department of Clinical Neurosciences University of Oxford Oxford UK; ^3^ Mental Health Neuroscience Department, Division of Psychiatry and Max Planck UCL Centre for Computational Psychiatry and Ageing Research University College London London UK; ^4^ Department of Clinical and Movement Neurosciences University College London London UK; ^5^ Wellcome Centre for Human Neuroimaging University College London London UK; ^6^ Department of Neurology Surgery University of California San Francisco San Francisco USA; ^7^ Department of Neurosurgery University of California San Francisco San Francisco USA

**Keywords:** beta burst prediction, subthalamic nucleus, Parkinson's disease, deep brain stimulation

## Abstract

**Background:**

The corticobasal ganglia network in Parkinson's disease (PD) is characterized by the occurrence of transient episodes of exaggerated beta frequency oscillatory synchrony, known as bursts. Although it is known that bursts of prolonged duration associate closely with motor impairments, the mechanisms leading to burst initiation remain poorly understood. Related to this, current adaptive deep brain stimulation (DBS) approaches reactively deliver stimulation following burst onset but cannot stimulate proactively to prevent bursts from occurring. The discovery of predictive biomarkers could allow for proactive stimulation, thereby offering potential for improvements in therapeutic efficacy.

**Objectives:**

We aimed to address this issue, by using deep neural networks to discover features of basal ganglia activity that reliably precede beta burst onset.

**Methods:**

We developed a deep learning model to predict burst onset from subthalamic nucleus (STN) activity recordings in PD patients. Our model provides patient‐specific predictions in two independent datasets of STN recordings, including prolonged‐duration recordings from sensing‐enabled DBS devices during naturalistic behaviors.

**Results:**

The occurrence of STN beta bursts can be reliably predicted up to 100 ms prior to onset. Importantly, our results reveal that a dip in the beta amplitude—which is likely to be indicative of a phase reset of oscillatory populations occurring between 80 and 100 ms prior to burst onset—is a predictive biomarker for burst occurrence.

**Conclusions:**

These findings demonstrate proof‐of‐principle for the feasibility of beta burst prediction and inform the future development of intelligent DBS approaches with the capability of proactive stimulation to prevent beta burst occurrence. © 2025 The Author(s). *Movement Disorders* published by Wiley Periodicals LLC on behalf of International Parkinson and Movement Disorder Society.

## Introduction

Parkinson's disease (PD) is characterized by nigrostriatal dopamine depletion and the emergence of stereotyped patterns of oscillatory synchrony within corticobasal ganglia circuits.^1^, ^2^ Excessive synchronization across the beta frequency range (13–30 Hz) is believed to relate closely to dopamine depletion and contributes to motoric impairment.^3^, ^4^ Therapeutic approaches such as subthalamic nucleus (STN) deep brain stimulation (DBS) and dopaminergic medication lead to a suppression of basal ganglia beta activity, with the extent of suppression correlating positively with motor improvements.^5^, ^6^, ^7^, ^8^, ^9^ Furthermore, a causal effect of beta oscillations on movement is highlighted by the observation that entraining motor cortical beta rhythms results in movement slowing.^10^

Recent observations highlight that beta activity is not continuous but occurs in short‐lived packets known as bursts.^11^, ^12^ Although the mechanisms of beta burst generation remain poorly understood, it is increasingly believed that bursts of longer duration and amplitude may be detrimental to motor function in PD.^13^ This finding has led to beta activity being used as a control signal in amplitude‐responsive closed loop DBS, where stimulation is delivered only when beta amplitude rises above a certain threshold.^14^, ^15^, ^16^, ^17^ Studies reveal that beta‐triggered adaptive DBS (aDBS) is more effective than conventional continuous DBS.^15^, ^17^, ^18^, ^19^, ^20^ Additionally, by virtue of selectively targeting a pathophysiological signal of interest, aDBS may offer additional benefits including reduced stimulation requirement and a lower incidence of stimulation‐induced side effects such as dyskinesia, gait impairment, and speech impairment.^21^

One drawback of aDBS is that stimulation is initiated after some fixed delay following the occurrence of a burst.^17^ This delay, which can be up to hundreds of milliseconds, will be the sum of the time taken for the burst to be detected and the system delay between burst detection and stimulation initiation.^17^ Consequently, aDBS can only reactively suppress beta bursts after they have developed and propagated within corticobasal ganglia circuits. The discovery of a reliable biomarker that could allow for the prediction of bursts would facilitate the development of proactive DBS approaches with the capability of either preventing bursts or suppressing them earlier following their onset.^22^ Proactive DBS approaches could lead to reduced stimulation requirement—as more energy tends to be required to reverse an established oscillatory state than to prevent it^23^—and consequently to improvements in both the efficacy and the side effect profile of DBS.

In this study we tested the hypothesis that STN activity carries neurophysiological features which reliably predict beta burst onset. We developed a deep neural network that models burst prediction as a binary classification problem. Our network can provide patient‐specific predictions from STN local field potential (LFP) recordings of beta band activity, which is known to be a robust motor biomarker for motor function in aDBS applications.^12^, ^17^, ^24^, ^25^

## Methods

### Patients and Experimental Details

We studied patients from two surgical centers: (1) the National Hospital for Neurology and Neurosurgery, University College London (UCL), UK and (2) the University of California, San Francisco (UCSF), USA. Data from UCL were collected during the acute postoperative period, whilst data from UCSF were wirelessly streamed from a sensing‐enabled DBS device several weeks after surgery. Clinical details for each patient are provided in Table S1. All patients provided written informed consent and research protocols were approved by the local research ethics committees at both sites.

### UCL Dataset

STN activity was recorded from 16 patients with PD who underwent bilateral implantation of STN DBS electrodes. In all cases, a Medtronic model 3389 electrode with four platinum‐iridium contacts was implanted. Recordings were performed 3–6 days after electrode implantation, before connection and insertion of the implantable pulse generator. Further details of the surgical procedure can be found in other reports.^26^, ^27^ PD diagnoses were made in accordance with the Queen Square Brain Bank Criteria.^28^

To maximize the probability of beta burst occurrence, recordings were performed following overnight withdrawal from dopaminergic medication (OFF medication). LFP activity was collected using a power main optically isolated BrainAmp system (Brain Products) with a sampling frequency of 2400 Hz.^5^, ^29^ Three bipolar channels (formed from contacts 0–1, 1–2, and 2–3; with contacts labeled anatomically in order from inferior to superior) were recorded from each electrode and the data were subsequently high‐pass filtered at 1 Hz in the hardware to avoid amplifier saturation due to large direct current (DC) offsets.

Patients were requested to keep their eyes open and to remain still during recordings. Either one or two rest recording sessions were performed. The duration of each session varied between 188 and 253 s (with a mean and standard error of the mean [SEM] of 198 ± 4 s; see Table S1).

### UCSF Dataset

Data were analyzed from five patients with PD, each with bilateral implants of the Summit RC + S neural interface (Medtronic). Each STN was implanted with a quadripolar Medtronic 3389 lead and data from two bipolar STN channels (formed from contacts 0–2 and 1–3) was wirelessly streamed from each hemisphere (sampling rate 250 Hz) to a Microsoft Windows tablet.^16^, ^30^ In all cases, recordings were completed between 2 and 4 weeks after implantation, whilst patients engaged in activities of daily living on their usual doses of dopaminergic medication. During this period, therapeutic DBS had not yet been initiated. In total this dataset included over 600 hr of STN activity recorded during naturalistic behaviors (see Table S1 for further details of recording durations for each patient).

For some of the data from each hemisphere, motor symptoms were monitored concurrently using Personal KinetiGraph® (PKG®) devices worn on both wrists (Global Kinetics Pty Ltd). These devices provided measurements of bradykinesia, tremor, and dyskinesia every 2 min, that were synchronized with contralateral neural recordings using timestamp alignment as previously described.^16^, ^30^, ^31^ Bradykinesia scores were first transformed from negative to positive, before being used to classify periods of sleep, using immobility as a surrogate marker (at least 6 min of immobility with a bradykinesia score of >80 within each 10‐min window).^30^, ^31^ Similarly, neural data corresponding to bradykinesia scores of <80 were classified as belonging to the awake state.

**FIG. 1:**
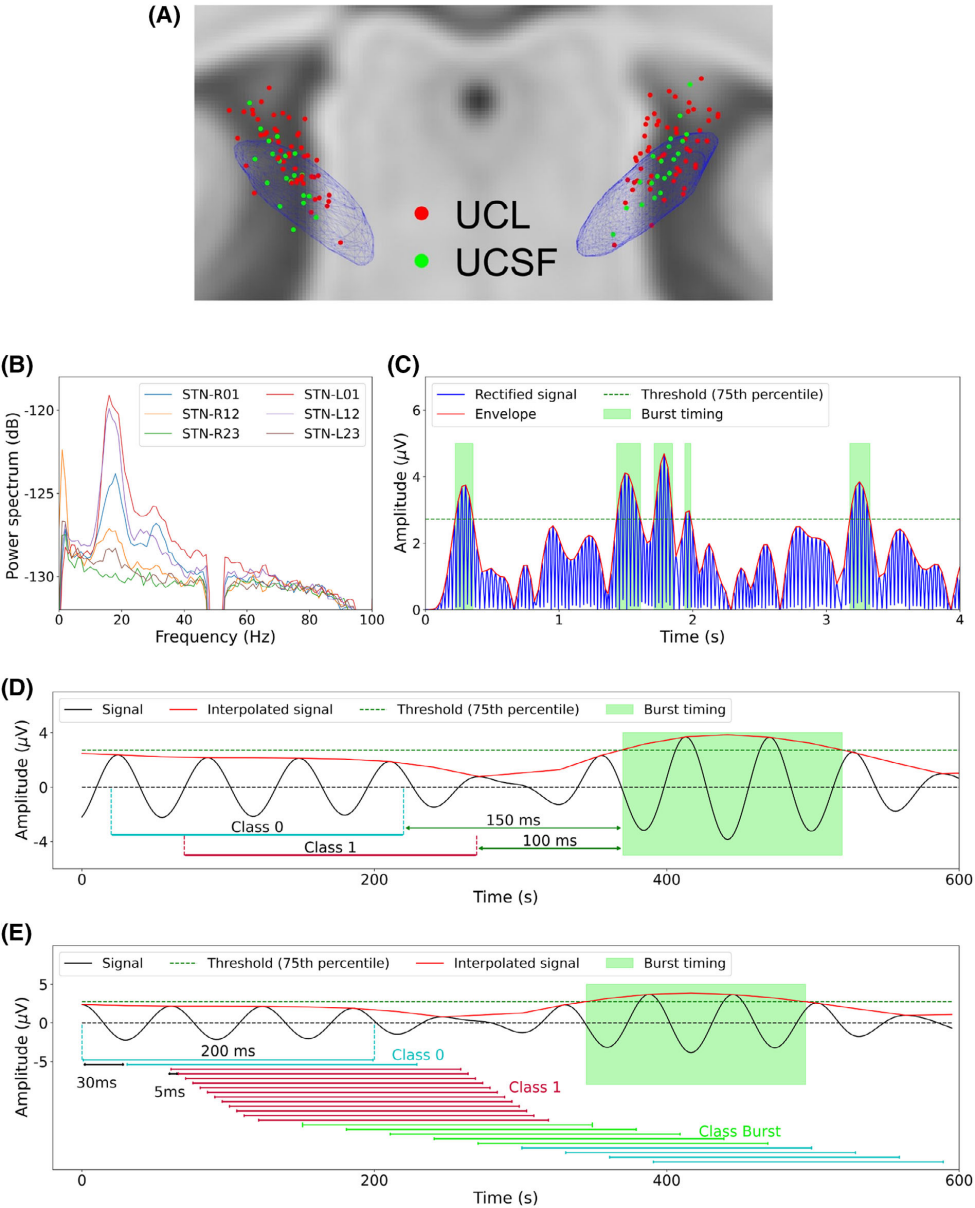
Electrode contact locations, labeling of beta bursts, and data segmentation for training the prediction model. (A) Subthalamic nucleus (STN) electrode contact locations for all patients (University College London [UCL] patients in red and University of California, San Francisco [UCSF] patients in green) are superimposed on an STN mesh (blue) and template magnetic resonance imaging (MRI) (coronal view) viewed in Montreal Neurological Institute (MNI) space. (B) Exemplar power spectra of right and left hemisphere STN local field potential (LFP) signals (recorded from bipolar contact pairs 01, 12, and 23) from patient 5 of the UCL cohort are displayed. In this case, channel STN‐R01 from the right hemisphere and channel STN‐L01 from the left hemisphere provided the highest amplitude peak within the beta frequency range (peak frequencies at 18 and 16 Hz) and were therefore selected as the beta channels for the corresponding hemispheres for further analysis. (C) A 4 s long segment of data from the right hemispheric channel STN‐R01 of patient 5 is displayed. Data were first filtered (±3 Hz) around the beta peak frequency before being rectified and interpolated. The 75th percentile of the interpolated signal amplitude distribution (dashed green line) was used as a threshold to define the onset and offset of beta bursts (burst timings are shown in the green rectangular boxes). (D) Illustration of the fixed window approach, where 200 ms long data segments ending at fixed time intervals (0, 20, 40, 60, 80, 100, or 120 ms) prior to the onset of a burst are labeled as being predictive (Class 1) of subsequent burst onset. Non‐predictive data segments (Class 0) terminated at least 150 ms prior to the onset of a burst. (E) Demonstration of the sliding window approach. A 200 ms long window with a stride length of 30 ms was passed along the beta filtered time series. Twelve windowed segments with a shortened stride length of 5 ms (see main article text) were labeled as being predictive of subsequent burst occurrence (Class 1). Windows that ended during the occurrence of a burst (Class Burst) were excluded from subsequent analysis, whilst the remaining data segments were labeled as being non‐predictive (Class 0). [Color figure can be viewed at wileyonlinelibrary.com]

For all patients, STN contact locations were confirmed by linearly co‐registering postoperative imaging (computed tomography [CT] at UCSF; magnetic resonance imaging [MRI] at UCL) with a preoperative 3 T MRI as previously reported.^16^, ^27^ Group visualization was then performed by normalizing electrode contact locations into Montreal Neurological Institute (MNI) space using the Lead‐DBS toolbox^32^ (see Fig. 1A).

### Determination of Beta Peak Frequency and Beta Bursts

The power spectrum of the STN LFP from each bipolar contact was obtained using the short‐time Fourier transform (STFT). A Hamming window with a length of 1 s and an overlap of 50% was used for spectral estimation. The squared magnitude of the resulting complex spectrum was computed and averaged across all time windows for visualization between frequencies of 1 Hz and 100 Hz (resolution of 1 Hz; see Fig. 1B for exemplar spectrum). For each hemisphere, we selected the bipolar STN contact pair whose power spectrum exhibited the largest peak within the beta (13–30 Hz) frequency range. We refer to this pair as the beta channel.

LFP time series from the beta channel were bandpass filtered within a ± 3 Hz window centered on the beta peak frequency, using a causal sixth order Butterworth filter implemented in the SciPy library for Python (https://scipy.org/). The application of a causal filter served to prevent future samples from impacting current or past filter outputs which would be used for prediction. We limited our analysis to individual patient beta band filtered signals as these are typically used in aDBS implementations.^17^, ^25^ Note that the filtered UCL data were downsampled to 600 Hz to reduce computational cost. The data were rectified prior to the peak values being linearly interpolated to produce the beta amplitude envelope of the signal. Finally, beta burst timings were defined as time points where the beta amplitude envelope exceeded its 75th percentile^11^, ^12^ (see Fig. 1c). Burst characteristics (number, durations, and rate) are listed for each hemisphere separately in Table S1.

### Beta Burst Prediction Network: Architecture, Training, and Evaluation

LFP data from each hemisphere were divided into training, validation, and test subsets. The training data were used to develop candidate models, while the validation set was employed to refine model architecture and optimize hyperparameters (eg, learning rate and the sigmoid threshold for classification). Model performance was ultimately evaluated using the test dataset.

In the UCL dataset, for patients with two recording sessions, one session was designated solely for training, whilst the other was equally split between validation and test datasets (50% each). For patients with only a single recording session, data were partitioned with 70% allocated to training, and the remaining 30% evenly divided between validation and testing (15% each).

For recordings from UCSF, 70–80% of the data were used for training, 10–15% for validation, and the remaining 10–15% for testing. Importantly, training, validation, and test datasets were drawn from recordings collected on different days (which meant that the precise percentages for training, test, and validation splits could vary slightly for each hemisphere). This approach served to improve model generalization in time for each patient. For burst prediction during sleep and awake states, only UCSF recordings with concurrent PKG data were used. In this analysis, 80%, 10%, and 10% of the data were allocated to training, validation, and testing, respectively, without constraining the split by recording day.

The prediction network, illustrated in Figure S1, was constructed using a convolutional neural network (CNN) architecture implemented in TensorFlow, using the Keras application programming interface (API) (https://www.tensorflow.org and https://keras.io/api/). This choice of model was partly inspired by previous CNN‐based networks for electroencephalography (EEG) classification.^33^ Further details of the network architecture and training are provided in the Supplementary Methods.

### Labeling of Data for Neural Network Classification

To train and test the prediction network, we segmented beta‐filtered STN time series using fixed and sliding window approaches. The fixed window approach assessed prediction performance at fixed intervals before burst onset. In this approach, data segments ending at fixed time points (0, 20, 40, 60, 80, 100, and 120 ms) before burst onset were labeled as being predictive (Class 1) (see Figure 1D). For data from UCL, we used 200 ms long data segments, whilst 400 ms long data segments were constructed for data from UCSF. The optimal length of data segments was selected based on prediction performance obtained from validation datasets. To create non‐predictive (Class 0) segments, data ending ≥250ms before burst onset (excluding periods coinciding with burst timing) were segmented into 200 ms long (UCL dataset) or 400 ms long (UCSF dataset) epochs with a stride length of 50 ms.

The sliding window approach was designed to mimic real‐time burst prediction to control the timing of stimulation delivery. As before, the choice of sliding window length and stride length was made empirically based on performance metrics from the validation datasets. After testing four different window lengths (200, 250, 300, and 400 ms) and seven stride lengths (10, 20, 25, 30, 35, 40, and 50 ms), we found that a 200 ms long window length with stride length of 30 ms resulted in optimal performance for UCL data, whilst a window length of 400 ms and a stride length of 50 ms provided optimal performance for UCSF data. Note that the sampling rates of the two datasets differed, meaning that the length of the input data (in samples) was similar for both datasets.

Figure 1E shows an example of the sliding window approach for data from UCL. If the end of a windowed segment overlapped with the occurrence of a burst, that segment was excluded from subsequent analysis (see green segments labeled Class Burst in Fig. 1E). For training the network to learn burst predictive features, the final three data segments occurring prior to each burst were subsampled with a smaller stride length of 5 ms, yielding 12 200 ms long data segments that terminated within 30–90 ms of burst onset (referred to as Class 1). This smaller stride length served to expand (augment) the size of the training dataset and to allow the network to be sensitive to data features that exhibit subtly variable timing (smoothness) in relation to burst onset. The remaining segments were associated with the absence of a subsequent burst and were labeled as being non‐predictive (referred to as Class 0 in Fig. 1E). For the fixed window approaches the number of Class 0 segments was matched to the number of Class 1 segments to maintain balance. However, for the sliding window approach, all segments were included in the training, validation, and test datasets.

Classification of data segments that included burst occurrence (Class Burst in Fig. 1E) was not deemed essential in our framework, as burst occurrence can be readily detected in real‐time using the burst amplitude threshold. Consequently, data segments corresponding to Class Burst were excluded from the prediction model.

### Evaluation of Burst Prediction Performance

The performance of the prediction model was quantified using the following metrics:

- True‐positives (TP): this is the number of burst predictive data segments (Class 1) that were correctly identified.
- True‐negatives (TN): this is the number of non‐predictive data segments (Class 0) that were correctly identified.
- False‐positives (FP): this refers to non‐predictive data segments (Class 0) where the model incorrectly predicted subsequent burst occurrence.
- False‐negatives (FN): this refers to predictive data segments (Class 1), where the model failed to predict subsequent burst occurrence.
- False‐positives per minute (FP/min): this quantifies the rate of FP occurrence.
- Accuracy (ACC): this indicates the proportion of correct predictions and is defined as: ACC = (TP + TN)/(TP + TN + FP + FN).
- Precision (PRC): tells us the proportion of correct positive predictions and is defined as: PRC = TP/(TP + FP).
- Sensitivity (SEN): also known as recall, this illustrates how well the model predicts the occurrence of bursts and is defined as: SEN = TP/(TP + FN).
- Area under the receiver operating characteristic curve (AUC‐ROC)—this metric considers the trade‐off between sensitivity and FP rate at various thresholds. It provides a measure of the classifier's ability to correctly distinguish between classes and was used for the fixed window approach where class membership was balanced.
- Area under the precision‐recall curve (AUC‐PR)—this considers the trade‐off between precision and sensitivity at various thresholds and is therefore not influenced by a disproportionately high occurrence of TN predictions.^34^, ^35^ The AUC‐PR was used instead of the AUC‐ROC for the sliding window approach, owing to its increased effectiveness for classification evaluation on imbalanced datasets.
- Prediction time prior to burst occurrence (PT‐PBO)—this metric was computed for the sliding window approach as the mean time interval between the prediction time and the start of a burst

## Results

### STN Beta Bursts Can be Predicted in Advance of their Onset

**FIG. 2:**
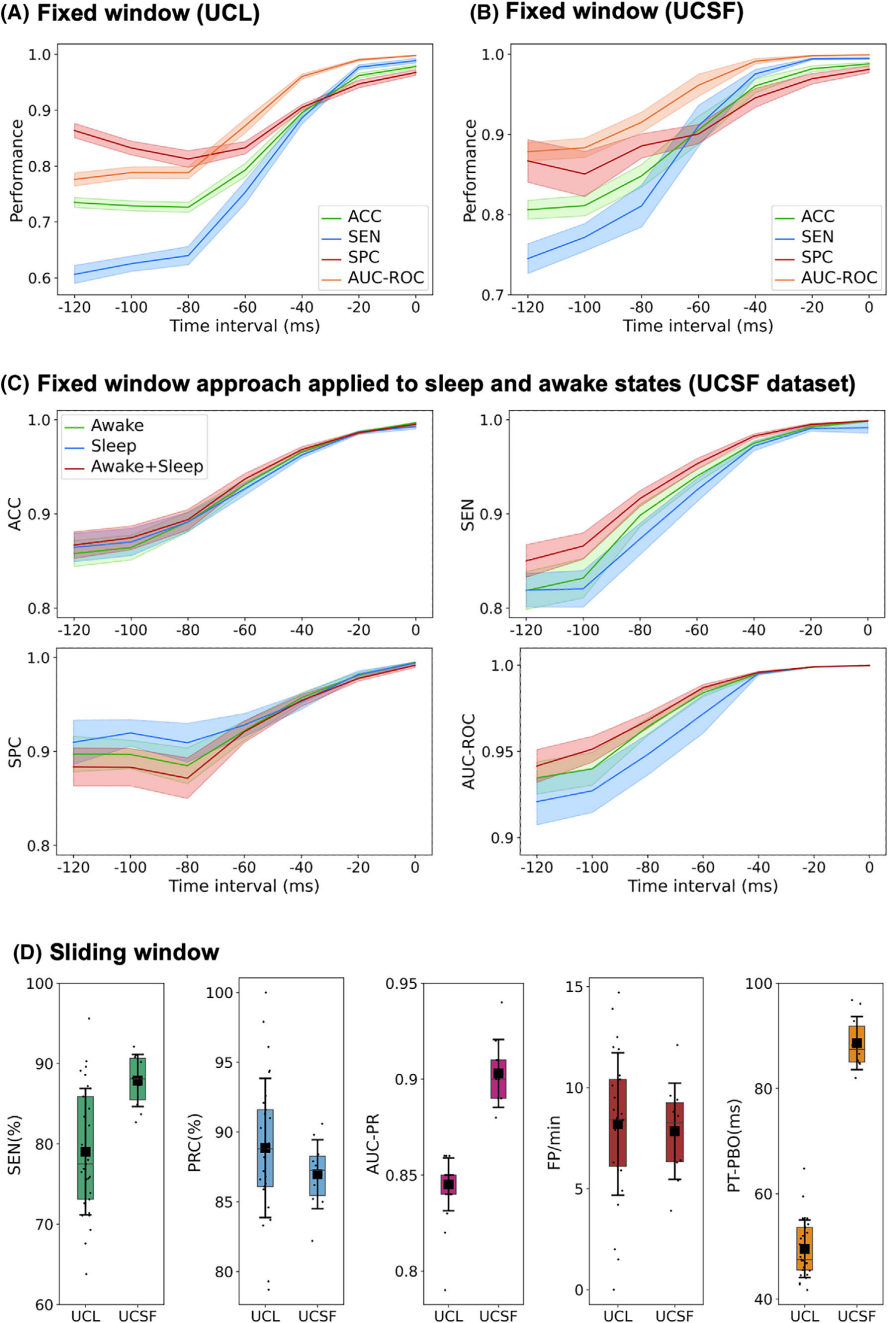
Burst prediction performance metrics for the fixed window and sliding window approaches. Burst prediction performance metrics for the fixed window approach are shown separately for the University College London (UCL) (A) and University of California, San Francisco (UCSF) (B) datasets. Averaged performance metrics across patients (accuracy [ACC], sensitivity [SEN], specificity [SPC], and area under the ROC curve [AUC‐ROC]), are illustrated for the seven fixed predictive window termination timepoints relative to burst onset. Solid lines indicate mean values, with shaded areas representing the standard error of the mean. Prediction performance metrics remain high as early as 60 ms before burst onset for the UCL data and as early as 100 ms prior to burst onset for the UCSF data (see main Results for further discussion). (C) Burst prediction performance is shown for the UCSF dataset, segmented according to the following physiological states: (1) the awake state, (2) the sleep state, and (3) combined data from the sleep and awake states. Performance metrics (ACC, SEN, SPC, AUC‐ROC) were comparable across all three conditions, indicating that model performance was not affected by behavioral state. (D) Shows burst prediction performance metrics for the sliding window approach for both the UCL and UCSF datasets. Values from each hemisphere of SEN, precision (PRC), area under the precision‐recall curve (AUC‐PR), and false‐positive rate (FP/min). SEN is plotted against FP/min, for each patient's prediction model, separately for FP/min, and PT‐PBO are demonstrated. Colored boxes highlight the interquartile range (IQR), with the mean indicated by a black square. The black whiskers represent the standard deviation, illustrating variability in burst prediction performance across patients. The results are in keeping with those from the fixed window approach, such that prediction performance metrics remain high as early as 50 ms before burst onset for the UCL data and 90 ms before burst onset for the UCSF data. [Color figure can be viewed at wileyonlinelibrary.com]

For the fixed window approach, we were able to achieve high burst prediction performance up to 100 ms before burst onset for the longer UCSF dataset. For shorter data from UCL, prediction performance metrics remained high up to 60 ms before burst onset. Importantly, data from UCSF were wirelessly streamed during activities of daily living and therefore provided a longer and more comprehensive training set for deep learning—potentially leading to improved performance. This information is summarized in Figure 2A,B, which show mean and standard error values for ACC, SEN, SPC, and AUC‐ROC across patients at seven different predictive window termination timepoints relative to burst onset (0, 20, 40, 60, 80, 100, and 120 ms) for the UCL (a) and UCSF (b) datasets. Performance metrics for individual patients are also detailed in Tables S2–S5 for the UCL data and Tables S6–S9 for the UCSF data. We also evaluated burst prediction performance separately for the awake and sleep states for the UCSF dataset, using the fixed window approach. Prediction models were trained, validated, and tested independently for sleep and awake data, and also on a combined dataset including both states. The results of this analysis are shown in Figure 2C and reveal that prediction performance is comparably high for the physiological states of sleep and wakefulness.

Prediction metrics for the sliding window approach applied to data from both centers are shown in Figure 2D. For the UCSF dataset, our model achieved a high SEN of 88% and a mean prediction time (PT‐PBO) of approximately 90 ms—corresponding closely to the result obtained using the fixed window approach. For the shorter duration UCL data, the SEN was 79%, with a PT‐PBO of approximately 50 ms. Individual patient and hemisphere prediction performance metrics for the sliding window approach are presented in Tables S10 (UCL dataset) and S11 (UCSF dataset).

Importantly, the inference time for our trained models (using TensorRT‐based inference) for a single data segment was typically less than 0.2 ms, highlighting potential for real‐time applicability.

### Classification Threshold Controls Trade‐Off between Sensitivity and FP Rate

**FIG. 3:**
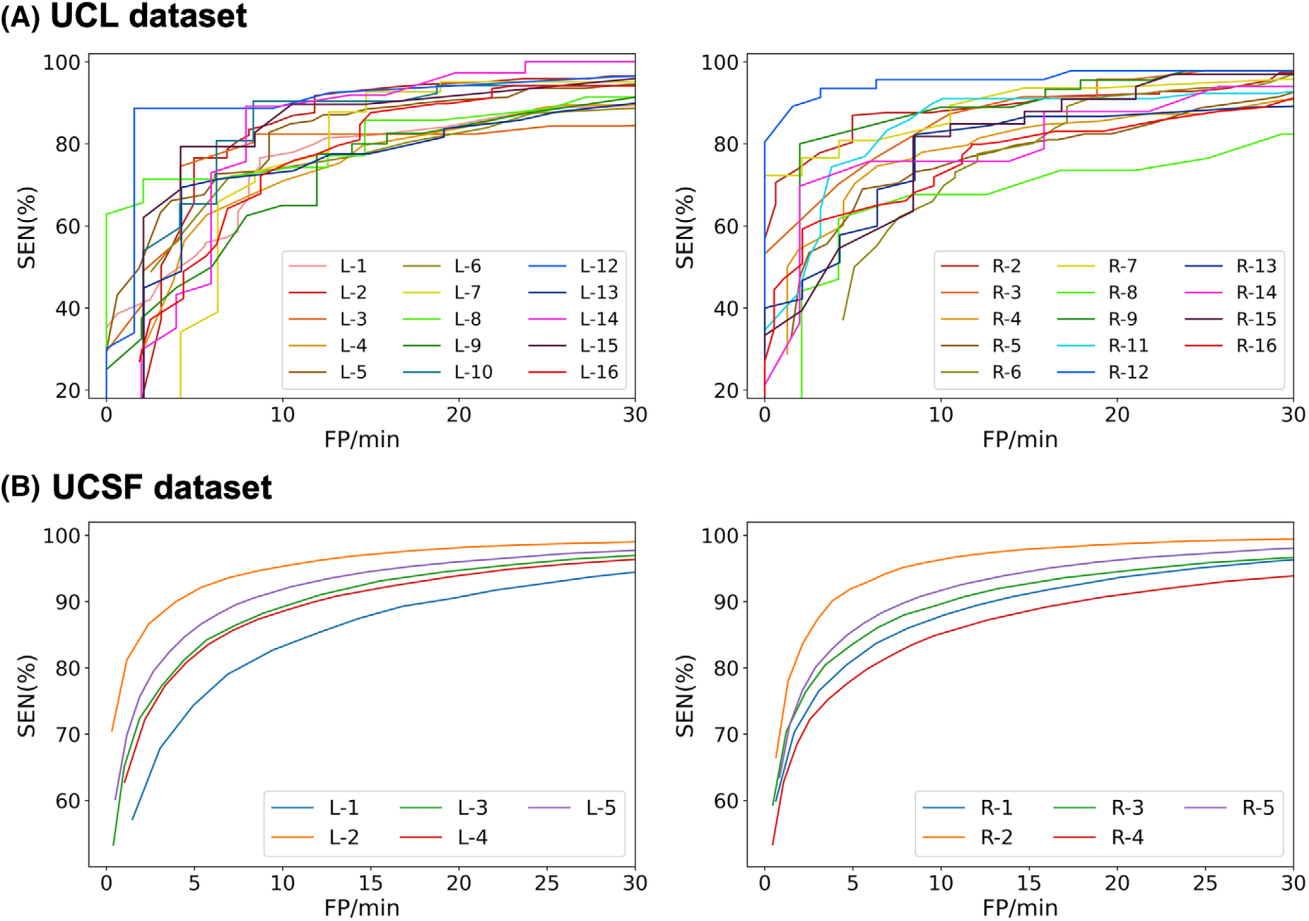
Classification threshold and the trade‐off between sensitivity (SEN) and false‐positive rate (FP/min). SEN is plotted against FP/min, for each patient's prediction model, separately for left (left panels) and right (right panels) hemispheric subthalamic nucleus (STN) channels, for the sliding window approach. Results are shown separately for the University College London (UCL) (A) and University of California, San Francisco (UCSF) (B) datasets. Each colored line represents a different patient. Lowering the classification threshold increases SEN, at the cost of an increased FP/min. Note that the left hemisphere of subject 11 and the right hemisphere of subjects 1 and 10 in the UCL dataset were excluded from analysis due to lack of a clear beta peak in the power spectrum. [Color figure can be viewed at wileyonlinelibrary.com]

An ideal burst prediction model should have a high SEN and a low FP/min. The trade‐off between these two performance metrics is determined by the sigmoid classification threshold of the output layer of the neural network, which binarizes predictions. Figure 3 shows the effect of varying the classification threshold on the SEN and FP rate for each patient's burst prediction model, for the sliding window approach. It is seen that lowering the threshold results in increased sensitivity at the cost of an increased FP rate. For the longer UCSF data, higher sensitivities are obtained for a given FP rate.

### Beta Amplitude Modulations Precede Subsequent Burst Occurrence

Our results reveal that beta burst occurrence may be accurately predicted as early as 100 ms before burst onset. But is there a consistent data feature allowing for this prediction which tells us about the neurophysiological origins of beta bursts? To address this, we examined mean beta amplitude envelopes for test data segments leading to TP, FP, and FN predictions. This procedure was performed separately for each hemisphere from each patient. Results are presented for the sliding window approach. In the case of TP and FN predictions, we selected the latest predictive data segment preceding the burst.

**FIG. 4:**
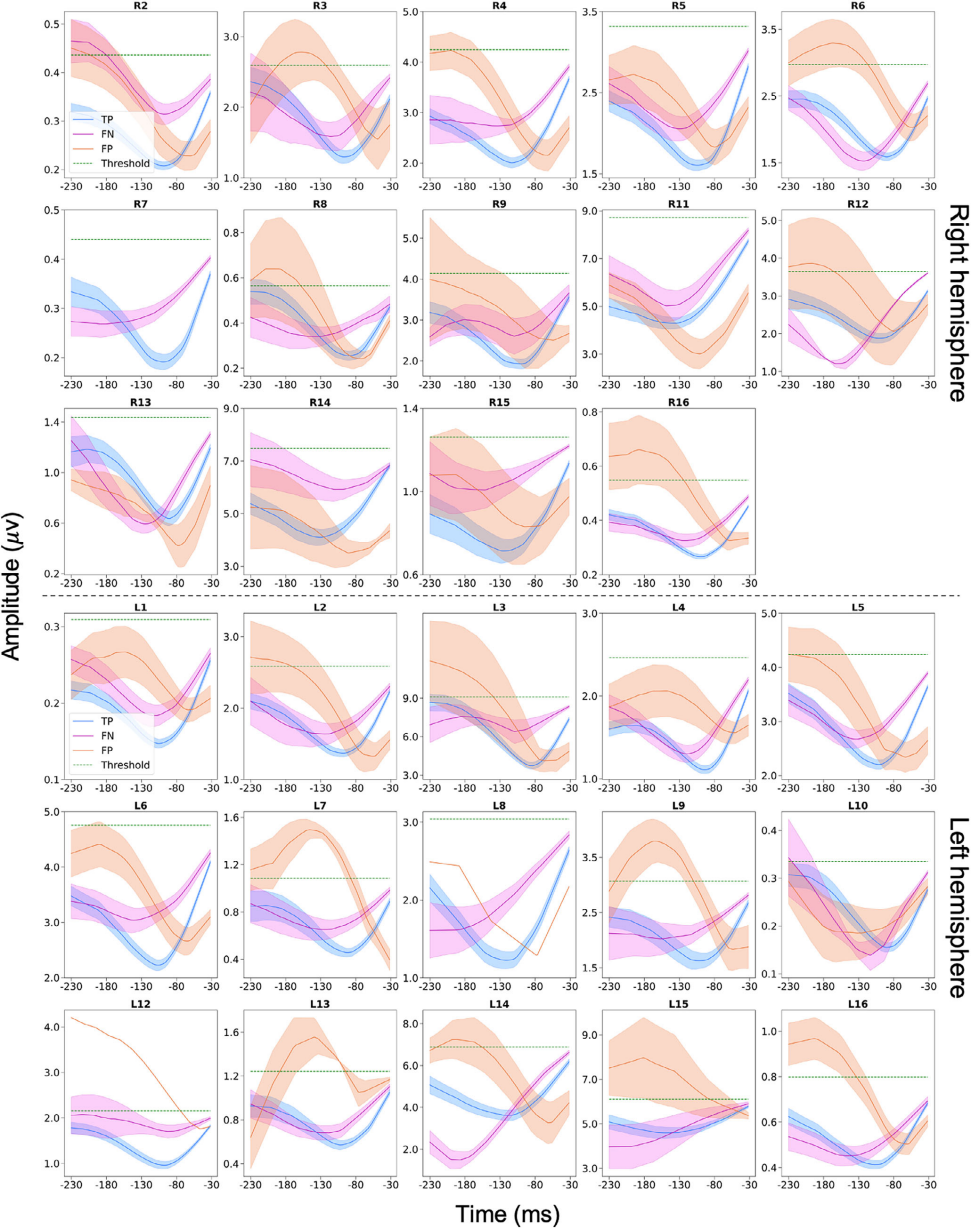
University College London (UCL) data: beta activity exhibits a characteristic dip 80–100 ms prior to burst onset. Data are shown separately for the right (upper panel) and left (lower panel) hemispheres. Mean individual patient beta envelope amplitudes for (200 ms long) data segments leading to true‐positive (TP), false‐negative (FN), and false‐positive (FP) predictions with the sliding window approach are shown. TP and FN predictions are synchronized to burst onset, whereas FP predictions are not, as they can occur at any time relative to the burst. Shaded areas represent the standard error of the mean. For each patient the amplitude threshold (75th percentile) for defining burst occurrence is also indicated by the green dashed line. For the right hemisphere of patient 7 there were no FP predictions. The left hemisphere for subject 11 and the right hemisphere for subjects 1 and 10 did not show a clear beta peak in the power spectrum and were therefore excluded from further analysis. [Color figure can be viewed at wileyonlinelibrary.com]

**FIG. 5:**
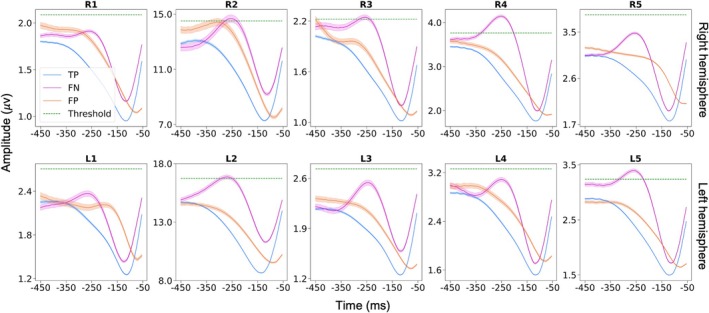
University of California, San Francisco (UCSF) data: beta activity exhibits a characteristic dip 80–100 ms prior to burst onset. Data are shown separately for the right (upper panel) and left hemispheres (lower panel). Mean individual patient beta envelope amplitudes for (400 ms long) data segments leading to true‐positive (TP), true‐negative (TN), and false‐positive (FP) predictions with the sliding window approach are shown. TP and false‐negative (FN) predictions are synchronized to burst onset, whereas FP predictions are not, as they can occur at any time relative to the burst. Shaded areas represent the standard error of the mean. For each patient the amplitude threshold (75th percentile) for defining burst occurrence is also indicated by the green dashed line. [Color figure can be viewed at wileyonlinelibrary.com]

Figures 4 and 5 reveal the results of this analysis separately for the UCL and UCSF datasets. Crucially, the plots reveal that for TP predictions there is a consistent dip in the beta amplitude across patients, which occurs approximately 80–100 ms prior to beta burst onset. Note that in these figures the predictive window ends either 30 ms (UCL data) or 50 ms (UCSF data) prior to burst onset. As expected, the beta amplitude profile of FP predictions was similar to that of TP predictions. Interestingly for FN predictions (which were on the whole rare; see Tables S10, S11), there was often a trend for the beta amplitude to rise above the burst threshold, before a subsequent dip that was less pronounced in amplitude than the dip exhibited by TP predictions.

In order to determine whether a simple beta dip detection algorithm could effectively predict burst onset for each hemisphere, we performed an additional analysis for the UCSF dataset. A beta dip template was derived from the mean beta envelope of the training dataset for Class 1 trials in the fixed window approach (with the window terminating at burst onset, corresponding to 0 ms). The Pearson correlation coefficient was then computed between this template and the beta envelope of Class 0 and Class 1 trials in the validation and test datasets. A threshold correlation coefficient for binary classification was derived from the validation dataset and applied to the test dataset, enabling us to derive prediction performance metrics for a beta dip detection algorithm. Figure S2 shows the distribution of the Pearson correlation coefficient values for Class 0 and Class 1 trials in the test dataset for each hemisphere. Mann–Whitney U tests (see Table S12 for test statistics) revealed that the distributions for Class 0 and Class 1 trials were significantly different for each hemisphere, with Class 1 trials exhibiting a larger proportion of high correlation values as expected. Despite this, however, prediction performance metrics for the beta dip detection algorithm were only modestly above chance and not at the level of the neural network‐based approach (see Table S12). Although our results reveal that a dip in the beta amplitude is a reproducible physiological feature occurring before bursts, simple algorithms for burst prediction based on dip detection perform less well than our CNN approach. This is likely to reflect the ability of CNNs to capture a richer repertoire of predictive dynamics than can be achieved using simple linear correlation‐based approaches.^36^

Finally, we sought to validate our findings using surrogate data. Further details of this analysis can be found in the Supplementary Results.

## Discussion

Our study demonstrates that the occurrence of pathological beta bursts within the parkinsonian STN can be accurately predicted from preceding STN beta activity. By training patient‐specific deep learning models, we show that bursts can be predicted with high precision up to 100 ms prior to their onset. Importantly, we demonstrate that burst prediction can be accurate using short amounts of training data, averaging less than 4 min for each patient. Furthermore, using prolonged recordings from sensing‐enabled DBS devices during activities of daily living, we illustrate the feasibility of burst prediction across naturalistic pathophysiological states (eg, during off periods, movement and dyskinesias^16^, ^30^). Finally, our analysis highlights that a dip in the beta amplitude, which occurs between 80 and 100 ms prior to burst onset, is a consistent predictive feature of beta burst occurrence.

These results provide proof‐of‐principle for the feasibility of burst prediction and also provide insights into the neurophysiological mechanisms leading to burst initiation. The advent of sensing‐enabled DBS devices^15^, ^37^ offering capabilities for adaptive stimulation and the deployment of portable machine learning algorithms (eg, TinyML^38^) offers significant future scope for implementing personalized stimulation approaches, such as the one presented here.

### Translational Potential of Burst Prediction

Beta frequency oscillatory activity within the parkinsonian corticobasal ganglia circuit has proven to be a robust biomarker of motoric impairments—particularly bradykinesia and rigidity.^4^, ^7^, ^31^, ^39^ Moreover, aDBS approaches which involve the delivery of STN stimulation only after the detection of beta bursts are more effective in terms of achieving motor benefit and limiting stimulation‐related side effects than conventional continuous DBS.^15^, ^17^, ^21^, ^40^ Although aDBS has been shown to be effective, a key drawback of beta amplitude‐triggered approaches is that they cannot prevent the onset of pathological beta bursts but rather respond with stimulation delivery after some fixed delay following burst onset. Consequently, aDBS may be acting too late to optimally prevent the initiation and propagation of pathological oscillatory activity within the corticobasal ganglia circuit (akin to closing the stable door after the horse has bolted). This drawback speaks to the utility of patient‐specific burst prediction approaches which allow for the earlier detection of bursts, and consequently the earlier delivery of stimulation to facilitate their termination.

Although our findings provide proof‐of‐principle, further real‐time clinical testing is required to compare the efficacy (and side effect profile) of our approach to existing adaptive DBS algorithms. Interestingly, our approach draws important parallels with prior work exploring seizure forecasting from intracranial recordings.^41^, ^42^, ^43^ CNNs have proven to be successful in this regard, as they allow for the automatic discovery of salient neurophysiological features,^36^ whereas simpler classical machine learning approaches (eg, logistic regression) typically require feature prespecification. Our CNN‐based approach can offer rapid predictions (<0.2 ms) for real‐time applications and may also be leveraged to predict other oscillatory features relating to distinct cognitive and motor phenotypes of PD.^30^, ^44^, ^45^

### Insights into Network Mechanisms Leading to Burst Generation

A key finding of our work is that the beta amplitude displays a stereotyped pattern of modulation prior to the onset of bursts. Figures 4 and 5 reveal the nature of this modulation, with the beta amplitude initially falling, reaching a nadir at approximately 80–100 ms prior to burst onset, and then subsequently rising again towards burst initiation. But what are the neural mechanisms underlying this phenomenon? Computational models relying upon stochastic inputs to neuronal populations as a primary driver of bursting cannot easily recapitulate the pre‐burst amplitude dip.^22^, ^46^, ^47^ Usually in such models, noisy inputs determine transitions of the system between stable (non‐oscillatory) and unstable (oscillatory) states via a bifurcation, meaning that oscillatory amplitudes can only change in one direction (increase or decrease). A more plausible explanation for the beta amplitude dip relates to the communication through coherence hypothesis, which proposes that maximal oscillatory entrainment within a network requires optimal phase alignment of interacting oscillatory populations.^48^, ^49^, ^50^ Switching to an optimal phase alignment for burst initiation requires a coordinated phase reset of oscillatory populations within the corticobasal ganglia circuit, which could account for the transient beta amplitude decreases.^50^

### Study Limitations

Our findings should be interpreted in light of the following limitations. Firstly, we predicted the occurrence of beta bursts regardless of their duration. Meanwhile, recent work has suggested that only bursts of prolonged duration (>200 ms) are likely to be associated with motor impairment in PD.^11^ Importantly however, we have separately shown that the same neural network architecture can be trained to predict whether a burst will be long or short within 25 ms of its onset,^51^ thus offering a time saving of up to 175 ms. Secondly, we have predicted STN beta bursts from STN activity and have therefore not included nodes within the corticobasal ganglia circuit—such as primary motor cortex (M1)—whose activities may facilitate STN burst prediction.^22^, ^52^ Although electrocorticographic recordings from M1 are currently performed only for research purposes, there is growing evidence to suggest that these may provide complementary information about clinical states (eg, dyskinesia).^30^

## Author Roles

(1) Research Project: A. Conception, B. Organization, C. Execution; (2) Statistical Analysis: A. Design, B. Execution, C. Review and Critique; (3) Manuscript Preparation: A. Writing of the First Draft, B. Review and Critique.

B.A.‐S.: 1A, 1B, 1C, 2A, 2B, 2C, 3A, 3B.

S.S.: 1B, 1C, 2A, 2B, 2C, 3B.

A.S.: 1B, 1C, 2C, 3B.

T.L.: 1C, 2C, 3B.

A.G.: 1C, 2C, 3B.

H.A.: 1C, 2C, 3B.

L.Z.: 1C, 2C, 3B.

P.L.: 1C, 2C, 3B.

T.F.: 1C, 2C, 3B.

T.D.:1C, 2C, 3B.

H.T.: 1C, 2C, 3B.

V.L.: 1C, 2C, 3B.

S.L.: 1A, 1B, 1C, 2C, 3B.

P.A.S.: 1A, 1B, 1C, 2C, 3B.

A.O.: 1A, 1B, 1C, 2A, 2B, 2C, 3B.

## Financial Disclosures

T.D. is a founder, director, and shareholder of Amber Therapeutics Ltd, which also has a controlling interest in Bioinduction Ltd and Finetech Medical Ltd. T.D. is also an advisor for Synchron and Cortec Neuro. P.S. is a consultant for Neuralink and InBrain Neuroelectronics. S.L. is a consultant for Iota Biosciences. The other authors declare no competing interests.

## Supporting information


**Data S1.** Supporting Information.

**Data S2.** Supporting Information.

## Data Availability

Anonymized datasets used for the current study are available from the corresponding authors on request and will be deposited on the MRC Brain Network Dynamics Unit data‐sharing platform (https://data.mrc.ox.ac.uk/data-set/). Python code for network training and evaluation is available at https://github.com/b-abdi/Burst-Prediction/tree/main.
