# Supplementary material for: Prediction of Pathological Subthalamic Nucleus Beta Burst Occurrence in Parkinson's Disease
Source: Mov Disord. 2025 Oct 3;40(12):2615–27. doi: 10.1002/mds.70076 (PMC12710186; doi:10.1002/mds.70076)
Supplement: Supplementary file 1 — Data S1. Supporting Information. [file MDS-40-2615-s001.docx]

Supplementary Materials

**Supplementary Methods**

## **Beta burst prediction network: architecture and training**

The LFP signal was passed through a sequence of 1D convolution, followed by a rectified linear unit (ReLU) activation function and 1D max pooling. This process was repeated three times to capture deep temporal features. The output from the final max pooling layer was flattened and connected to a dense layer, before a sigmoid function was used to perform binary classification (yielding an output of 0 or 1, corresponding to each of the classes). A kernel size of 5 was used for the 1D convolution layers, to capture local time series features prior to burst onset. A pooling size of 2 was used for the 1D max pooling layers to half the temporal dimensionality. The dense layer employed a dropout rate of 50%. The network was trained to minimize the binary cross-entropy loss function, using the Adam optimizer with an initial learning rate of 0.0001 ^1^. Training used a maximum of 50 epochs, with a batch size of 8. Early stopping was employed based on the validation loss, with a patience of 5. All experiments were performed on a workstation equipped with an NVIDIA RTX 3090 GPU.

**Supplementary Results**

## **Validation of findings using surrogate data**

We sought to validate our findings, by generating surrogate data that preserved beta burst characteristics of the original data whilst destroying pathophysiological signal properties occurring within non-bursting time periods. We expected to see that this manipulation would lead to significantly impaired burst prediction performance metrics when training and testing our burst prediction network on surrogate data.

The original signal from each patient and hemisphere was bandpass filtered (using a zero-phase 6th order Butterworth filter with pass band of ±3 Hz around the beta peak frequency) to define periods of bursting activity. A surrogate signal was then created by preserving the original data from periods of bursting activity and randomly shuffling (in time) the data belonging to non-bursting time periods. The surrogate timeseries were then processed with the same causal filter used for the corresponding original dataset. A sliding window approach - as previously described - was employed to train and test the prediction network. The results of this analysis are presented in **Supplementary Table 13**. As anticipated, the model achieved poor performance compared to the original data, indicating that the network relies on the physiological signatures present in the original data to predict bursts, and that the narrow-band filter has no effect on network training.

**Supplementary Figures**


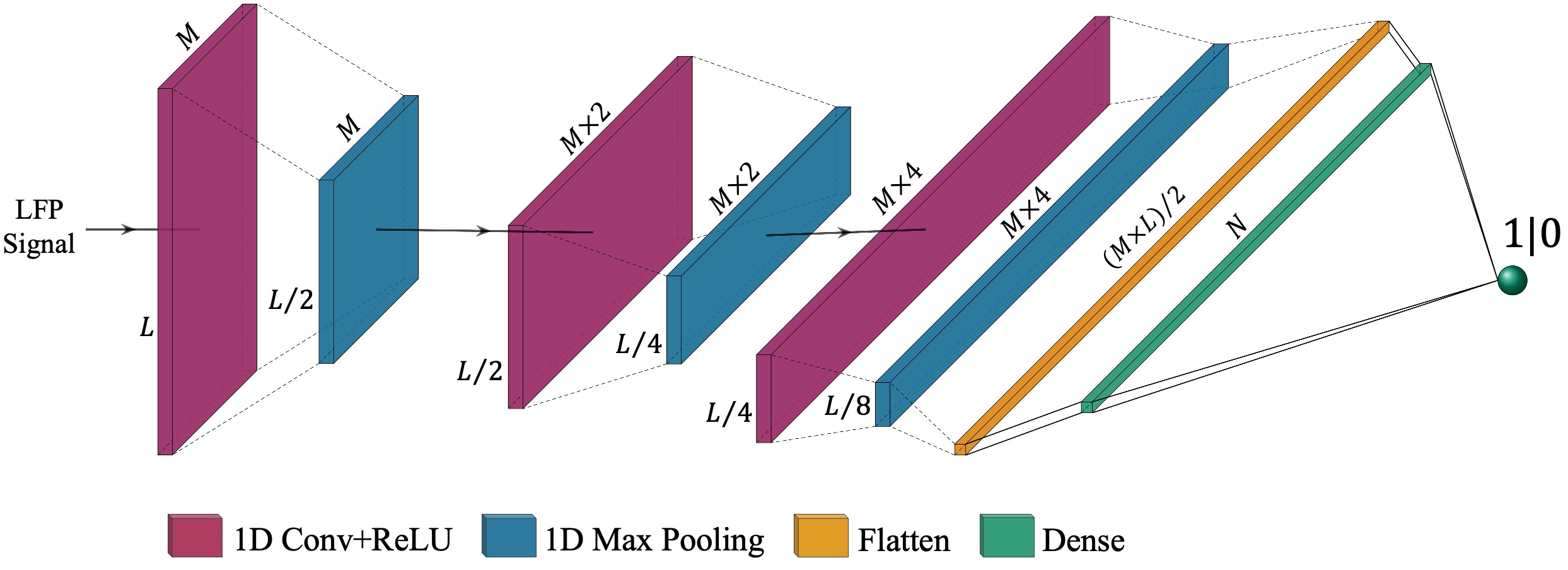


**Supplementary Figure 1. Architecture of the burst prediction network**. The input beta filtered LFP is passed through a sequence of Conv1D, ReLU, and Max pooling (see main text). This process is repeated thrice, before the resulting outputs are flattened and passed to a dense layer. A single unit is then used for binary classification using a sigmoid activation function. Here, $L$ is the length of input data (number of temporal features), which was 120 for the UCL data and 100 for the UCSF data. $M$ represents the number of filters in the convolutional layers and was selected to be 128. $N$ indicates the number of neurons in the dense layer, $N=512$.


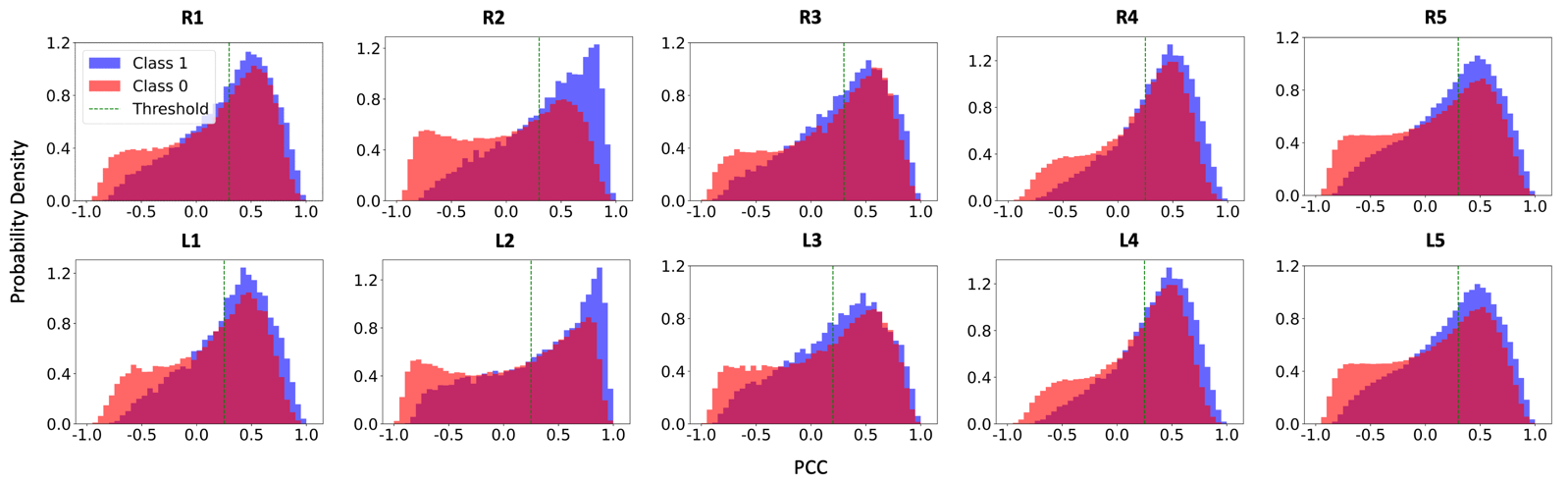
 **Supplementary Figure 2. Correlation of beta dip templates for burst prediction (UCSF dataset).** Probability density histograms of the Pearson correlation coefficient (PCC) between the beta dip template and the beta envelope of trials belonging to Class 0 or Class 1 test data segments are shown. Data are shown for each hemisphere of the UCSF dataset for the fixed window approach (window terminating at 0 ms). The vertical line marks the PCC threshold, derived from validation data, used for burst prediction. It can be seen that Class 1 trials have a greater proportion of high PCC values (see **Results** section for further details of this analysis).The vertical line marks the PCC threshold used for burst prediction.

**Supplementary Tables**

| Data | Case | Age Gender | Disease duration (years) | Preoperative medication (mg) | UPDRS III pre-op off/on | Data length UCL(s) S1/S2 UCSF(hrs) R/L | No. of bursts R/L | Mean burst duration (ms) R/L | Burst rate  (per second) R/L |
| --- | --- | --- | --- | --- | --- | --- | --- | --- | --- |
| **UCL** | 1 | 54/F | 10 | LDE 1158 | 35/9 | 192/193 | 667/623 | 126.6/123.3 | 1.7/1.6 |
|  | 2 | 54/M | 15 | LDE 1150 | 53/19 | 190/193 | 545/509 | 148.3/173.3 | 1.4/1.3 |
|  | 3 | 58/F | 3 | LDE 390 | 41/9 | 191 | 305/262 | 133.3/144.1 | 1.6/1.4 |
|  | 4 | 47/M | 8 | LDE 2264 | 46/4 | 190/188 | 500/559 | 128.3/126.6 | 1.3/1.5 |
|  | 5 | 57/M | 8 | LDE 1229 | 41/19 | 238/195 | 513/583 | 130.0/156.6 | 1.2/1.3 |
|  | 6 | 60/M | 27 | LDE 2048 | 63/8 | 194/189 | 615/597 | 136.6/131.6 | 1.6/1.5 |
|  | 7 | 57/M | 17 | LDE 1460 | 54/14 | 190 | 279/265 | 138.3/150.0 | 1.4/1.4 |
|  | 8 | 52/M | 13 | LDE 1484 | 35/10 | 191 | 225/241 | 148.3/151.6 | 1.1/1.3 |
|  | 9 | 58/M | 11 | LDE 1320 | 43/25 | 201 | 340/304 | 132.5/130.0 | 1.7/1.5 |
|  | 10 | 72/M | 9 | LDE 1281 | 28/5 | 192 | 316/307 | 127.5/130.0 | 1.6/1.6 |
|  | 11 | 60/M | 11 | LDE 1012 | 28/5 | 190/201 | 431/675 | 138.3/121.6 | 1.1/1.7 |
|  | 12 | 43/M | 9 | LDE 1650 | 63/40 | 253 | 346/340 | 134.1/150.8 | 1.3/1.3 |
|  | 13 | 41/M | 6 | LDE 1220 | 50/22 | 188 | 279/313 | 126.0/128.3 | 1.5/1.7 |
|  | 14 | 58/M | 12 | LDE 1500 | 38/14 | 202 | 282/250 | 140.8/141.6 | 1.4/1.2 |
|  | 15 | 60/M | 10 | LDE 1560 | 56/10 | 190 | 205/194 | 175.0/189.2 | 1.1/1.0 |
|  | 16 | 61/F | 11 | LDE 1049 | 35/4 | 192/225 | 626/566 | 119.1/150.0 | 1.5/1.3 |
|  | Mean$\pm$ SEM | 55.7$\pm$1.8 | 11.2$\pm$1.3 | 1361$\pm$ 107 | 44.3$\pm$2.8/ 13.5$\pm$2.4 | 198$\pm$4 | 404$\pm$38/ 411$\pm$41 | 136.4$\pm$3.2/ 143.6$\pm$4.6 | 1.4$\pm$0/ 1.4$\pm$0 |
| **UCSF** | 1 | 58/M | 12 | LDE 2100 | 44/22 | 58.6/18.9 | 198,140/72,429 | 175.8/156.9 | 0.94/1.0 |
|  | 2 | 54/M | 7 | LDE 1425 | 49/26 | 33.3/48.7 | 97,950/130,390 | 201.2/241.8 | 0.81/0.7 |
|  | 3 | 63/M | 19 | LDE 955 | 45/30 | 40.4/32.8 | 109,787/84,158 | 198.9/184.6 | 0.75/0.71 |
|  | 4 | 28/F | 12 | LDE 1550 | 61/27 | 45.1/52.9 | 126,837/139,038 | 185.0/183.2 | 0.78/0.73 |
|  | 5 | 40/M | 4 | LDE 1314 | 41/30 | 178.5/126.3 | 512,585/294,840 | 165.2/182.6 | 0.80/0.65 |
|  | Mean$\pm$ SEM | 48.6$\pm$6.4 | 10.8$\pm$2.5 | 1468$\pm$ 186 | 48$\pm$3.5/ 27$\pm$1.5 | 71.2$\pm$27.1/ 55.9$\pm$18.6 | 209059$\pm$77884/ 144171$\pm$39790 | 185.2$\pm$6.8/ 189.8$\pm$6.8 | 0.81$\pm$0.03/ 0.75$\pm$0.06 |

**Supplementary Table 1. Clinical characteristics of patients and details of STN recordings from UCL and UCSF**. The total pre-operative UPDRS part III score is presented in the on and off medication states. S1 and S2 indicate duration of STN recordings for sessions 1 and 2, for UCL patients. Burst characteristics are presented separately for data from the right (R) and left (L) hemispheres. LDE represents the levodopa dose equivalent.

**Supplementary References**

1. Kingma DP, Ba JL. Adam: A Method for Stochastic Optimization. *3rd International Conference on Learning Representations, ICLR 2015 - Conference Track Proceedings*. Published online December 22, 2014.
